# Evaluation of intestinal biopsy tissue preservation methods to facilitate large-scale mucosal microbiota research

**DOI:** 10.1016/j.ebiom.2024.105550

**Published:** 2024-12-31

**Authors:** Nicola J. Wyatt, Hannah Watson, Gregory R. Young, Mary Doona, Ned Tilling, Dean Allerton, Andrea C. Masi, Tariq Ahmad, Jennifer A. Doyle, Katherine Frith, Ailsa Hart, Victoria Hildreth, Peter M. Irving, Claire Jones, Nicholas A. Kennedy, Sarah Lawrence, Charlie W. Lees, Robert Lees, Trevor Liddle, James O. Lindsay, Julian R. Marchesi, Miles Parkes, Nick Powell, Natalie J. Prescott, Tim Raine, Jack Satsangi, Kevin Whelan, Ruth Wood, Andrew King, Luke Jostins-Dean, R. Alexander Speight, Naomi McGregor, Christopher J. Stewart, Christopher A. Lamb

**Affiliations:** a Translational & Clinical Research Institute, Faculty of Medical Sciences, Newcastle University, Newcastle upon Tyne, United Kingdom; b Department of Gastroenterology, The Newcastle upon Tyne Hospitals NHS Foundation Trust, Newcastle upon Tyne, United Kingdom; c Newcastle Clinical Trials Unit (NCTU), Newcastle University, Newcastle upon Tyne, United Kingdom; d Department of Gastroenterology, Royal Devon University Healthcare NHS Foundation Trust, Exeter, United Kingdom; e Exeter Inflammatory Bowel Disease and Pharmacogenetics Research Group, University of Exeter, Exeter, United Kingdom; f Department of Gastroenterology, St Marks Hospital and Academic Institute, Gastroenterology, London, United Kingdom; g Department of Surgery and Cancer, Imperial College, London, United Kingdom; h Department of Gastroenterology, Guy's & St Thomas' NHS Foundation Trust, London, United Kingdom; i School of Immunology & Microbial Sciences, King's College London, London, United Kingdom; j Department of Histopathology, The Newcastle upon Tyne Hospitals NHS Foundation Trust, Newcastle upon Tyne, United Kingdom; k Institute of Genetics & Molecular Medicine, University of Edinburgh, Edinburgh, United Kingdom; l Edinburgh IBD Unit, Western General Hospital, NHS Lothian, Edinburgh, United Kingdom; m Research Informatics Team, Clinical Research, The Newcastle upon Tyne Hospitals NHS Foundation Trust, Newcastle upon Tyne, United Kingdom; n Department of Gastroenterology, Barts Health NHS Trust, The Royal London Hospital, London, United Kingdom; o Centre for Immunobiology, Blizard Institute, Barts and the London School of Medicine and Dentistry, Queen Mary University of London, London, United Kingdom; p Division of Digestive Diseases, Department of Metabolism, Digestion and Reproduction, St Mary's Hospital, Imperial College London, London, United Kingdom; q Department of Gastroenterology, Cambridge University Hospitals NHS Foundation Trust, Cambridge, United Kingdom; r Department of Gastroenterology, Imperial College Healthcare NHS Trust, London, United Kingdom; s Department of Medical and Molecular Genetics, King's College London, Guy's Hospital, London, United Kingdom; t Nuffield Department of Medicine, University of Oxford, Old Road Campus, Oxford, United Kingdom; u Department of Nutritional Sciences, King's College London, London, United Kingdom; v Kennedy Institute of Rheumatology, University of Oxford, Oxford, United Kingdom

**Keywords:** Inflammatory bowel disease, Precision medicine, Gut microbiome, Tissue microbiome, Tissue preservative reagents, Formalin fixed paraffin embedded

## Abstract

**Background:**

Large-scale multicentre studies are needed to understand complex relationships between the gut microbiota, health and disease. Interrogating the mucosal microbiota may identify important biology not captured by stool analysis. Gold standard tissue cryopreservation (‘flash freezing’) limits large-scale study feasibility. We aimed to compare gut microbiota in gold standard and pragmatic mucosal biopsy storage conditions.

**Methods:**

We collected endoscopic recto-sigmoid biopsies from 20 adults with inflammatory bowel disease. Biopsies were preserved using three methods: (i) flash freezing (most proximal and distal biopsy sites); (ii) nucleic acid preservative reagents (QIAGEN Allprotect®, Invitrogen RNA*later*™, and Zymo DNA/RNA Shield™); and (iii) formalin fixation with paraffin embedding (FFPE), which is used to preserve tissue for clinical histopathology within healthcare settings. Microbiota were sequenced on the MiSeq platform (V4 region, 16S rRNA gene).

**Findings:**

Tissue microbiota were consistent between most proximal and distal tissue suggesting any within-patient variation observed reflected storage condition, not biopsy location. There was no significant difference in alpha-diversity or microbial community profiles of reagent-preserved versus gold standard tissue. FFPE community structure was significantly dissimilar to other tissue samples, driven by differential relative abundance of obligate gut anaerobes; *Faecalibacterium*, *Anaerostipes* and Lachnospiraceae. Despite these differences, tissue microbiota grouped by participant regardless of preservation and storage conditions.

**Interpretation:**

Preservative reagents offer a convenient alternative to flash freezing tissue in prospective large-scale mucosal microbiota studies. Whilst less comparable, FFPE provides potential for retrospective microbiota studies using historical samples.

**Funding:**

10.13039/501100000265Medical Research Council (MR/T032162/1) and 10.13039/100007028The Leona M. and Harry B. Helmsley Charitable Trust (G-2002-04255).

**Research in context**

**Evidence before this study**

Increasing evidence highlights the role of the gut microbiota in human health and disease. Studies require multicentre large-scale collection of samples to ensure data has adequate statistical power and is generalisable to diverse human populations. Stool is high biomass and convenient to collect at scale. However, stool reflects the distal luminal gut microbiota and may fail to identify microbiota signals at the mucosal surface, interrogation of which may provide important biological insights that may be otherwise missed. The mucosal microbiota can be sampled by biopsy at the time of lower gastrointestinal endoscopy (colonoscopy or flexible sigmoidoscopy). Flash freezing is considered the gold standard for preserving microbiota integrity. However, this relies upon access to ultra-low temperature cold chain infrastructure that is likely to limit feasibility of large-scale multicentre studies. Accordingly, most studies to date have involved one or a small number of recruitment sites and thus are limited in power. Commercially available nucleic acid preservative reagents have been developed as an alternative to flash freezing and are in widespread research use for human cellular storage prior to gene expression assays. Formalin fixation with paraffin embedding (FFPE) is used routinely to preserve human tissue within healthcare settings in preparation for histopathological assessment and long-term clinical storage. To our knowledge, there is no published literature that has systematically evaluated these more pragmatic preservative conditions for mucosal gut microbiota research, comparing with gold standard flash freezing.

**Added value of this study**

In this study, we demonstrate that intestinal biopsies stored in three commercially available tissue nucleic acid preservative reagents (QIAGEN Allprotect®, Invitrogen RNA*later*™, and Zymo DNA/RNA Shield™) produced comparable microbiota signatures to one another and the accepted gold standard of flash freezing. Modelling pragmatic tissue handling conditions in multicentre research, we found that comparability of microbiota signatures was independent of temporary storage duration on wet ice/at 4 °C, and of final storage temperature (−80 °C versus −20 °C). Stool samples generated a different microbiota signal to tissue samples, supporting our own and others’ hypothesis that profiling the gut microbiota with stool may fail to capture important microbial signals at the mucosal surface.

We showed that mucosal microbiota can be interrogated with FFPE tissue, and that microbiota data can be bioinformatically corrected for contaminating reads using wax controls. Whilst the microbial community structures in FFPE were dissimilar to other conditions, tissue microbiota data grouped by participant across all conditions, including FFPE. This suggests that whilst a limited source of microbial data, microbiota features reflective of the participant persist in FFPE tissue.

**Implications of all the available evidence**

Mucosal microbiota studies employing 16S rRNA gene sequencing techniques are not compromised by adoption of tissue preservative agents. These preservatives may support much needed pragmatic clinical protocols for collection of biopsies at scale from multiple recruiting centres as part of well-powered prospective mucosal microbiota studies. With limitations, FFPE gut tissue which is commonly stored long-term by healthcare organisations after clinical procedures, provides potential for retrospective microbiota analysis.

## Introduction

The gastrointestinal tract is colonised by trillions of microorganisms, including bacteria, archaea, fungi and viruses. Collectively referred to as the gut microbiota, interest in this complex microbial ecosystem has grown exponentially in the last two decades. Encoding at least 100-fold more genes (collectively, the gut microbiome) than the human host genome,^1^ gut microbiota can produce short-chain fatty acids and essential nutrients, as well as contributing to biotransformation of drugs, conjugated bile acids and xenobiotics.^2^ As understanding of this metabolically active ‘organ’ has evolved, so too has interest in its role in the development of human disease. Through complex interactions with genetic susceptibility, immune, and environmental factors, the gut microbiota are thought to play a key role in the aetiopathogenesis of multiple conditions. This includes inflammatory bowel disease (IBD), a condition encompassing Crohn's disease and ulcerative colitis that is characterised by dysregulated host–microbe interactions and a diverse inflammatory burden.^3^^,^^4^

The gut microbiota is typically studied using stool, which is a relatively cheap and non-invasive sample to collect.^5^ Commercially available preservative kits enable stool sample collection and storage at room temperature for several weeks.^6^^,^^7^ This allows research participants to collect samples at home (remotely) and return these directly to research facilities via postal service. However, stool is not spatially resolved so cannot be used to assess longitudinal variation in microbial composition within the gastrointestinal tract.^8^ This lack of resolution is an important consideration given intra- and inter-individual variation in inflammation across different segments of the large and small intestine seen in patients with IBD. Stool may also fail to capture subtle microbial compositional changes at the mucosal surface or within physical niches such as colonic crypts.^9^ Interfacing the host immune system and gut microbiota, intestinal epithelial barrier dysfunction has long been implicated in IBD aetiopathogenesis.^10^^,^^11^ Evaluating the mucosal microbiota using tissue biopsy specimens may contribute to improved understanding of host–microbiota interactions at the mucosal surface, in both health and disease states.

To resolve which microbiota factors are implicated in IBD pathogenesis, including responsiveness to therapeutic interventions, large multi-centre studies are needed. The gold standard for preserving tissue samples for microbiota analyses is immediate flash freezing with long-term storage at ultra-low temperatures (e.g. −80 °C).^12^^,^^13^ Within multi-centre studies, access to cold chain infrastructure may not be available or may vary between participating research sites. Lack of standardised sample collection and handling workflows risks introduction of potential biases that confound downstream data analysis. Consequently, most tissue studies to date have involved one or a small number of recruitment sites and thus are limited. To facilitate successful delivery of standardised research across multiple sites, including cross-replication cohorts, gold standard approaches must be balanced with pragmatic protocols.

Several commercially available tissue reagents exist to preserve biological sample integrity without the need to store at ultra-low temperatures.^14^^,^^15^ These reagents are typically used for preservation of nucleic acids in human cellular samples prior to gene expression assays. Furthermore, within healthcare settings, formalin fixation with paraffin embedding (FFPE) is routinely used to preserve tissue morphology and cellular detail for histopathological assessment and diagnosis. These FFPE samples are stored at room temperature for several decades.^16^ To inform the design of future pragmatic, large-scale mucosal microbiota research protocols, we aimed to determine if microbiota signatures could be derived from reagent preserved and FFPE intestinal biopsies comparing with gold standard (flash frozen) tissue samples.

## Methods

**Fig. 1:**
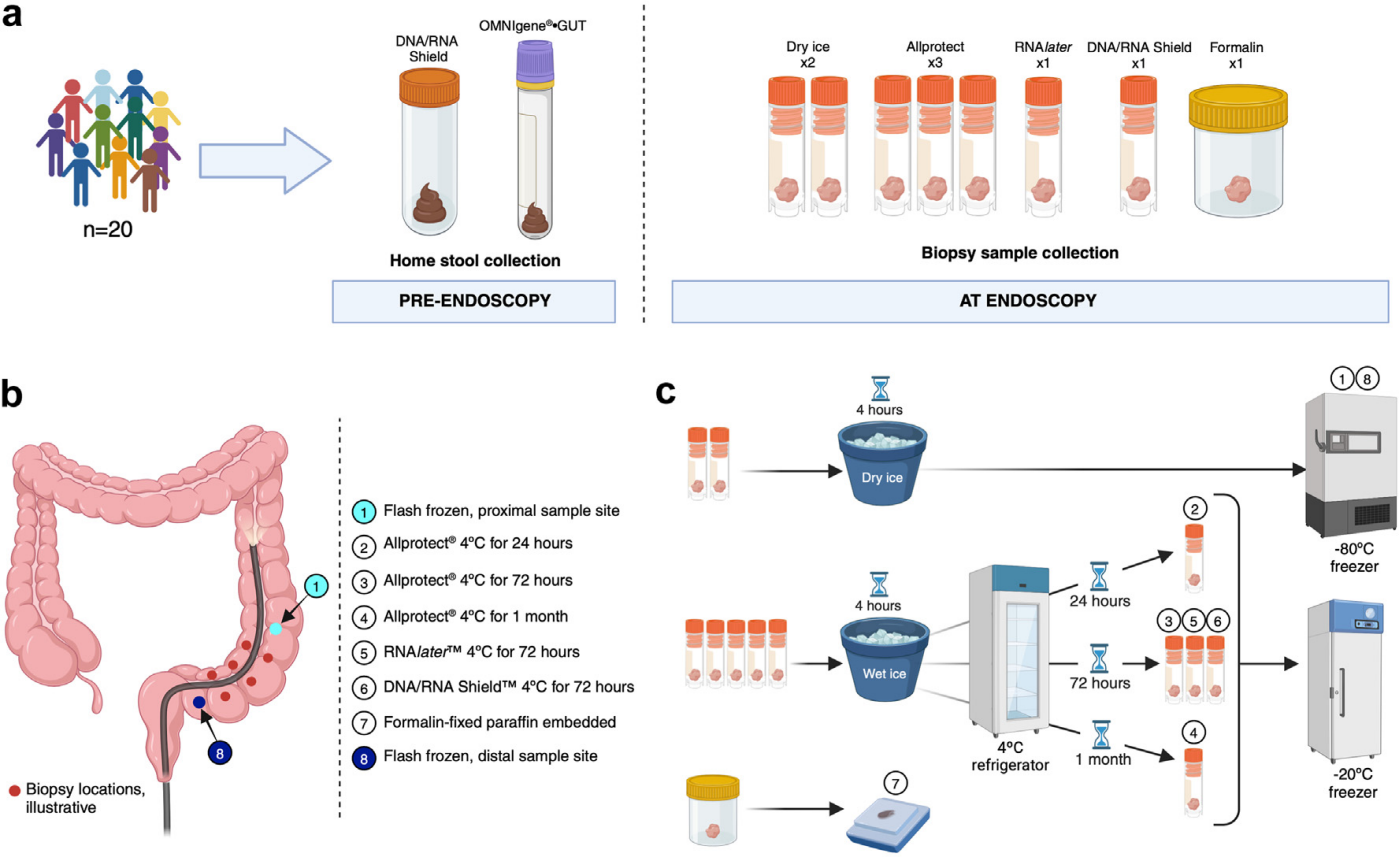
**Schematic summarising study sample collection and biopsy processing**. (a) Participant samples collected during the study (2 stool and 8 tissue samples per participant); (b) Illustrative locations and storage conditions for tissue biopsy samples collected from participants at lower gastrointestinal endoscopy procedures; (c) Overview of sample processing and storage conditions. Samples were immediately placed into pre-defined conditions within the endoscopy room. Conditions 1 and 8: Samples were flash-frozen with dry ice and transferred to −80 °C storage within 4 h. Conditions 2–6: Samples were placed into a cryovial pre-filled with one of three storage buffers before placing into wet ice for a maximum of 4 h before transfer to 4 °C. Samples remained at 4 °C for 24 h (Condition 2), 72 h (Conditions 3, 5 and 6) or 1 month (Condition 4) before transfer to −20 °C. Condition 7: Tissue sample placed into formalin pre-filled pot and sent to the Cellular Pathology Department at the Royal Victoria Infirmary, Newcastle upon Tyne Hospitals NHS Foundation Trust, for processing into a formalin-fixed paraffin embedded research block using the standard local laboratory protocol. Created in BioRender. Wyatt, N. (2024) BioRender.com/p99f243.

A schematic providing an overview of the study is provided in Fig. 1.

### Participants

We recruited 20 adult participants with a diagnosis of IBD (Crohn's disease, ulcerative colitis or IBD-unclassified) who were scheduled to attend the Royal Victoria Infirmary (RVI, part of The Newcastle upon Tyne Hospitals NHS Foundation Trust, NUTH) for a planned outpatient lower gastrointestinal endoscopy; colonoscopy (n = 18) or flexible sigmoidoscopy (n = 2) (Table 1). Participants were identified from endoscopy bookings and contacted by a member of the clinical team to discuss the study further. Those interested in taking part were pre-screened and sent a copy of the patient information sheet.

**Table 1:** Summary table of study participants and disease characteristics.

**Diagnosis**, n (%)	
Crohn's disease	8 (40%)
Ulcerative colitis	12 (60%)
**Sex**, n (%)	
Female	8 (40%)
Male	12 (60%)
**Age**, mean ± SD (years)	43.4 ± 15.7
**Disease duration**, mean ± SD (years)	17.7 ± 7.5
**Previous IBD surgery**, n (%)	6 (30%)
**Total PRO-2 score (1 day prior to bowel preparation)**, median [range]	
Crohn's disease	2.3 [0–7.75]
Ulcerative colitis	1 [0–4]
**Endoscopic assessment of disease activity**	
Crohn's disease: SES-CD, n (%)	
Remission (score 0–2)	4 (50%)
Mild (score 3–6)	1 (12.5%)
Moderate (score 7–15)	2 (25%)
Severe (score >15)	1 (12.5%)
Ulcerative colitis: Mayo endoscopic subscore, n (%)	
Normal/inactive (score 0)	7 (58%)
Mild (score 1)	2 (17%)
Moderate (score 2)	2 (17%)
Severe (score 3)	1 (8%)
**Exposure to biologic therapies**, n (%)	
Biologic naïve	8 (40%)
On first biologic	8 (40%)
On second biologic	4 (20%)
**Current use of biologic therapies**, n (%)	
Infliximab	5 (25%)
Adalimumab	2 (10%)
Vedolizumab	4 (20%)
Ustekinumab	1 (5%)
**Current use of other IBD therapies**, n (%)	
5-ASA	10 (50%) (as monotherapy, n = 5)
Azathioprine	4 (20%) (as monotherapy, n = 1)
Methotrexate	1 (5%) (as monotherapy, n = 0)

### Ethics

This research was conducted as a pre-planned methods development sub-study within IBD-RESPONSE (Defining microbial predictors of responsiveness to advanced therapies in Crohn's disease and ulcerative colitis).^17^ Ethical approval was obtained from the Wales Research Ethics Committee 5 (reference 21/WA/0228). All participants provided informed written consent prior to taking part in the study. Consent forms were signed by participants remotely via electronic consent forms embedded into a study-specific REDCap (Research Electronic Data Capture) online database. Participants accessed eConsent forms remotely using a web browser on a personal laptop, tablet or mobile device. Valid consent was verified by an appropriately delegated member of the research team prior to research participation.

### Stool sample collection

Following verification of participant consent, a stool collection kit was posted to participants at their home address. Participants were asked to collect two stool samples from a single bowel movement before commencing bowel preparation for their procedure (as close as possible to procedure date, maximum five days prior). Samples were collected into sterile containers pre-filled with preservative buffer: OMNIgene®•GUT kit (DNAGenotek, product code: OMR-200) and DNA/RNA Shield™ stool collection tube (Zymo, catalogue number: R1101). Each stool collection pack contained two DNAGenotek OMNIgene® toilet accessories (product code: OM-AC1) and two single-use spoons (product code: OM-AC2) to facilitate sample collection, along with participant instructions. Stool samples were stored at room temperature until the day of the scheduled endoscopy procedure. Upon receipt, stool samples were transferred to a −80 °C freezer until DNA extraction was performed. Nucleic acid extraction was performed for use in downstream sequencing applications, as described below.

### Endoscopy procedure and mucosal biopsy sampling

Endoscopy procedures were performed as part of planned hospital care with consent to obtain additional biopsies for research. Procedures were performed by the study authors (CAL, RAS and AK) using high-definition Olympus colonoscopes and 2.4 mm jaw biopsy forceps (Radial Jaw™ 4, Boston Scientific, catalogue no. M00513330). A total of eight recto-sigmoid biopsies were collected from each participant, in addition to standard sampling for routine clinical histopathological assessment. Sampling time and site data (distance from the anal verge) were recorded for each research sample collected. A clean pipette tip was used to transfer each biopsy sample from the biopsy forceps into a pre-labelled cryovial, minimising sample contact and risk of contamination. Biopsies from the most proximal and most distal sampling sites were subject to identical gold standard handling and storage (flash frozen using dry ice, with long-term storage at −80 °C) to ensure that any intra-individual difference observed between sample conditions reflected storage conditions and not variation by sampling site. All other cryovials were placed into wet ice in the endoscopy room for a maximum of 4 h, prior to transferring to refrigerator (4 °C) then freezer (−20 °C) storage (Fig. 1). FFPE biopsies were fixed in 10% formalin and embedded in paraffin at the RVI Cellular Pathology department. FFPE samples were processed in identical conditions to routine paraffin embedding under non-sterile conditions to ensure applicability of research results to clinically collected specimens.

In the IBD-RESPONSE study, we planned to use QIAGEN Allprotect® solution to preserve tissue biopsy samples collected during endoscopy procedures. Prior to archival storage at −20 °C or −80 °C, the manufacturer recommends that tissue is incubated in the reagent at 2–8 °C overnight. In this methods optimisation study, we evaluated samples stored in QIAGEN Allprotect® solution at 2–8 °C for 24 h, 72 h and one month, prior to transferring to −20 °C storage. These times were chosen to reflect: (a) the shortest anticipated time from sample collection at a participating research site to archival low temperature storage at a central receiving laboratory; (b) the realistic time taken to transfer samples from a participating research site to a central receiving laboratory, factoring in possible weekend delays in shipping; and (c) research design in which participating sites return samples to a central receiving laboratory in batches, with local storage at 4 °C, as a way of minimising complexity and cost of sample collection. To ensure that the QIAGEN Allprotect® solution was an appropriate choice for the IBD-RESPONSE study, we also evaluated tissue stored in two other commercially available preservative reagents: Invitrogen RNA*later*™ stabilisation solution (Fisher Scientific, product code: 10564445) and Zymo DNA/RNA Shield™ (Cambridge Biosciences, product code: R1100-50) (Fig. 1).

### DNA extraction

All DNA extraction procedures were performed in a sterile class II cabinet using sterile equipment. To minimise batch effects, all samples from a given participant were extracted together, typically alongside other participants. For tissue samples stored in preservative reagent, single-use sterile inoculation loops were used to retrieve samples and place these directly into DNA extraction kit PowerBead Pro tubes. Extraction kit negative controls were included with each batch.

#### Cryopreserved tissue, reagent-preserved tissue and stool samples

DNA was extracted from flash frozen and reagent-preserved biopsy specimens, and stool for downstream analysis using the QIAamp® PowerFecal Pro DNA Kit (QIAGEN, catalogue number: 51804) according to the manufacturer instructions with some modifications: CD1, 400 ml; CD2, 150 ml; CD3, 500 ml; C6, 40 ml. Mechanical disruption and homogenisation of samples was performed on the TissueLyser II system (QIAGEN, catalogue number: 5300; RRID:SCR_018623) with two shaking steps (25Hz for 5 min), rotating the tube rack between steps to ensure uniform disruption and homogenisation.

#### FFPE samples

DNA was extracted from FFPE biopsy specimens for downstream analysis using the QIAamp® DNA FFPE Advanced Kit (QIAGEN, catalogue number: 51804) according to the manufacturer instructions.

A 4 mm single-use punch biopsy tool was used to core biopsy specimens *en bloc*. Typical clinical histological processing of FFPE samples is not designed for downstream microbial sequencing and is therefore non-sterile. The tissue of interest is embedded within a block of wax that is a potential source of contamination. To control for this, a separate 4 mm wax-only core was taken from the FFPE block of each participant. DNA extraction was performed on wax cores, as for tissue, to assess for the presence of bacterial DNA in the wax itself and enable downstream bioinformatic controlling for this (see ‘Statistics’).

### 16S rRNA gene sequencing

Extracted nucleic acid concentrations were determined using a Qubit fluorimeter (Thermo Fisher, UK; RRID:SCR_018095), Supplementary Table S1.

Extracted nucleic acids were transported on dry ice to Baylor College of Medicine (Houston, Texas, United States) for 16S rRNA gene sequencing. Methods were adapted from the NIH-Human Microbiome Project,^18^^,^^19^ and the Earth Microbiome Project.^20^ The V4 region of the 16S rRNA gene was amplified and sequenced on the MiSeq (Illumina) platform using 2 x 250 bp paired-end reads. Read pairs were demultiplexed and reads quality filtered using bbduk.sh (BBMap, version 38.82).^21^ Illumina adapters, PhiX reads, reads with a Phred quality score (Q) < 15, and reads with length <100bp after trimming were removed. Reads were merged using bbmerge.sh with the following merge parameters: maxstrict = t, qtrim = t, trimq = 15 and filtered via vsearch (maximum expected error rate 0.05, minimum length 252bp, maximum length 254bp).^22^ Sequences were stepwise clustered into operational taxonomic units (OTUs) at a similarity cut-off value of 97% using the UPARSE algorithm.^23^ OTU centroids were mapped against the SILVA Database (version number 138.1, RRID:SCR_006423).^24^ Raw sequencing data is available at the European Nucleotide Archive under project accession PRJEB74359.

### Statistics

Analysis was performed in R (version 4.3.2, RRID:SCR_001905) using the phyloseq package (RRID:SCR_013080).^25^^,^^26^ Potentially contaminant taxonomic features were identified and removed using negative control sequencing data and the prevalence-based method (standard threshold = 0.1) within the decontam package in R.^27^ A list of taxa removed as potential contaminants (present in wax and/or kit negative controls) is available in Supplementary Table S2.

Samples with fewer than 1000 sequence reads following decontamination were removed from the analysis. All samples were rarefied prior to analysis to account for variable numbers of mapped bacterial reads across different sample types. Rarefaction depth was modified according to sample types being compared. A threshold of 1k reads was used to compare FFPE and any other sample type due to lower read depth in FFPE samples. All comparisons that did not include FFPE data used a threshold of 4.6k reads. Sufficient sampling depth following rarefaction was confirmed by visualising rarefaction curves (Supplementary Figure S1) and assessing Good's Coverage (Supplementary Figure S2 and Supplementary Table S3).

Alpha-diversity was calculated as taxonomic richness and Shannon diversity using the vegan R package (RRID:SCR_011950).^28^ Comparisons of continuous dependent variables, such as library size and alpha-diversity measures, were made using the Kruskal–Wallis rank-sum test and the pairwise-Wilcoxon test. Beta-diversity was calculated as weighted UniFrac dissimilarity. PERMANOVA was used to test significance of beta-diversity dissimilarity between groups. All p-values are adjusted for multiple comparisons with the Benjamini-Hochberg formula to control for the false discovery rate.^29^ Differential taxa between sample types were identified using the Multivariate associations with linear models (MaAsLin2) package for R (RRID:SCR_023241), using the sample type as a fixed effect and participant ID as a random effect.^30^ Data were visualised using the ggplot2 (RRID:SCR_014601),^31^ ggpubr (RRID:SCR_021139),^32^ and ggbiplot packages in R.^33^

### Role of funders

The funders had no role in the study design, data collection, data analysis or report writing. The decision to submit this manuscript for publication was made by the listed publication authors with no influence from the funders.

## Results

A total of 200 samples (intestinal biopsy, n = 160; stool, n = 40) from 20 adult participants with IBD (ulcerative colitis, n = 12; Crohn's, n = 8) and 40 negative control samples were analysed. Participant characteristics are summarised Table 1. Sex was defined as biological sex at birth. Stool samples were collected a mean of two days [IQR 1–2.25] prior to lower gastrointestinal endoscopy procedures (Supplementary Table S4). Random biopsy samples were collected with a median distance of 10 cm [IQR 8–11] between the most proximal and distal sampling sites (Supplementary Figure S3). Tissue preserved using FFPE remained in formalin for a median of one day [range 0–3] before embedding in paraffin wax (Supplementary Figure S4).

A total of 7,323,646 reads were obtained from 16S rRNA gene sequencing. One Zymo sample (participant B), and one FFPE sample (participant F) failed to amplify during the PCR step of sequencing. Gel electrophoresis showed high molecular weight smearing with no defined 16S band visible (data not shown). Read depth of successfully sequenced samples varied by sample type (Table 2). For one participant (participant F), the library size of all eight tissue samples was <1000 reads. This participant was excluded from the tissue microbiota analysis, along with FFPE samples from six other participants that had <1000 reads (participants C, E, K, L, P, T).

**Table 2:** Unrarefied library sizes by sample type prior to decontamination (samples with <1000 mapped reads excluded).

	Flash frozen proximal	Allprotect 4 °C 24 hr	Allprotect 4 °C 72 hr	Allprotect 4°C 1 month	RNAlater 4 °C 72 hr	Zymo 4 °C 72 hr	FFPE	Flash frozen distal	Stool
Samples, *n*	20	19	19	19	19	18	12	19	40
Total reads	893,670	924,895	1,041,2620	937,437	958,161	891,502	77,025	932,184	635,248
Median reads (IQR)	48,531 (43,493–52,266)	50,866 (41,813–53,023)	52,289 (46,334–58,828)	47,388 (42,461–54,505)	48,790 (44,782–53,561)	51,718 (44,136–58,044)	6615 (3819–9565)	48,491 (44,735–55,303)	16,071 (14,816–18,428)

OTUs identified as contaminants using the decontam package (28/982 taxa; 0.3% of the total reads) were removed from the dataset (Supplementary Table S2). After removing potentially contaminating taxa, library sizes of participant tissue (median = 48,609, IQR = 42,579–55,854) and stool (median = 16,071, IQR = 14,1812–18,428) samples were significantly greater than kit negative (median = 150, IQR 60–215) and wax (median = 523 IQR = 387–712) controls, P < 0.0001 (Kruskal–Wallis, Supplementary Figure S5). Community composition of participant samples was significantly dissimilar to kit negative controls and FFPE wax core controls (ANOSIM P < 0.05) (Supplementary Table S5, Supplementary Figure S6).

**Fig. 2:**
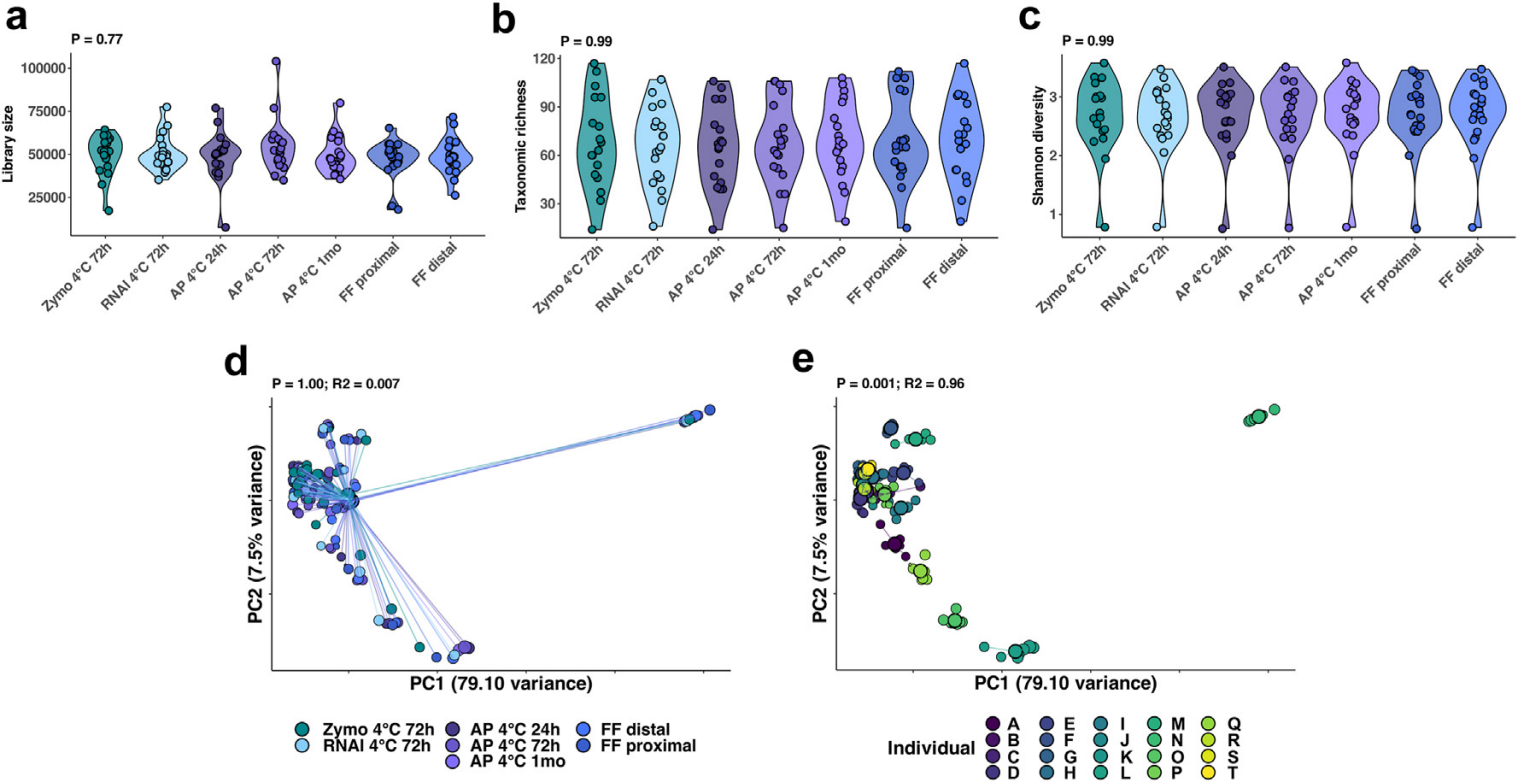
**Comparison of flash frozen (FF) and reagent-preserved tissue**. (a) Violin plots illustrate comparison of unrarefied library sizes (Kruskal–Wallis P = 0.77); (b) rarefied alpha-diversity (taxonomic richness) (Kruskal–Wallis P = 0.99); and (c) rarefied alpha-diversity (Shannon diversity index) (Kruskal–Wallis P = 0.99), where each point represents an individual sample. (d) Beta-diversity measures of bacterial community composition by preservation method (PERMANOVA P = 1, R^2^ = 0.007), and (e) participant (PERMANOVA P = 0.001, R^2^ = 0.55) are also shown. (Abbreviations: RNAl, RNAlater; AP, AllProtect).

The human gut microbiota changes along the longitudinal axis of the gastrointestinal tract.^16^^,^^34^ To ensure that any differences observed between tissue samples reflected the storage condition and not confounding by sampling site, the most proximal and distal biopsy samples were subjected to the same gold standard flash freezing conditions. Comparison of reagent-preserved tissue (conditions 2–5, Fig. 1) with gold standard samples identified no significant difference in mapped bacterial reads prior to rarefaction (Kruskal–Wallis P = 0.77, Fig. 2a) as well as rarefied tissue richness (Kruskal–Wallis P = 0.99, Fig. 2b), tissue diversity (Kruskal–Wallis P = 0.99, Fig. 2c), or microbiota composition (PERMANOVA P = 1, R^2^ = 0.007; Fig. 2d and Supplementary Figure S7). Differences in community composition were explained by the individual participant (PERMANOVA P = 0.001, R^2^ = 0.94; Fig. 2e).

Next, we sought to assess the impact of standard processing of fresh tissue into FFPE blocks by comparing microbiota profiles with other tissue, as well as stool. Having demonstrated no significant difference between the two flash frozen samples, all subsequent analyses including a flash frozen comparator were made using the distal sample data, for simplicity. Whilst FFPE samples yielded significantly greater unrarefied library sizes (median = 6,127, IQR = 3682–9328) than negative controls (pairwise-Wilcoxon P = 0.005, Supplementary Figure S8), libraries were significantly smaller than flash frozen tissue (median = 48,448, IQR = 44,722–55,236) (pairwise-Wilcoxon P = 4.3 × 10^−8^) (Table 2 and Supplementary Figure S9) and stool (median = 15,627, IQR = 14,932–17,622) (pairwise-Wilcoxon P = 3.0 × 10^−6^).

Both stool collection kits tested in this study yielded comparable results, with equal read depths (Kruskal–Wallis P = 0.62) and beta-diversity between samples (PERMANOVA P = 0.95, R^2^ = 0.009) (Supplementary Figure S10). As seen for FFPE tissue, library sizes for stool samples were significantly smaller than gold standard (pairwise-Wilcoxon P = 5.27 × 10^−9^) and reagent-preserved tissue (pairwise-Wilcoxon P = 2.2 × 10^−16^) (Supplementary Figure S10). Despite these differences, gold standard tissue and stool samples showed similar taxonomic richness (Kruskal–Wallis P = 0.32) and Shannon diversity (Kruskal–Wallis P = 0.14) (Supplementary Figure S10).

**Fig. 3:**
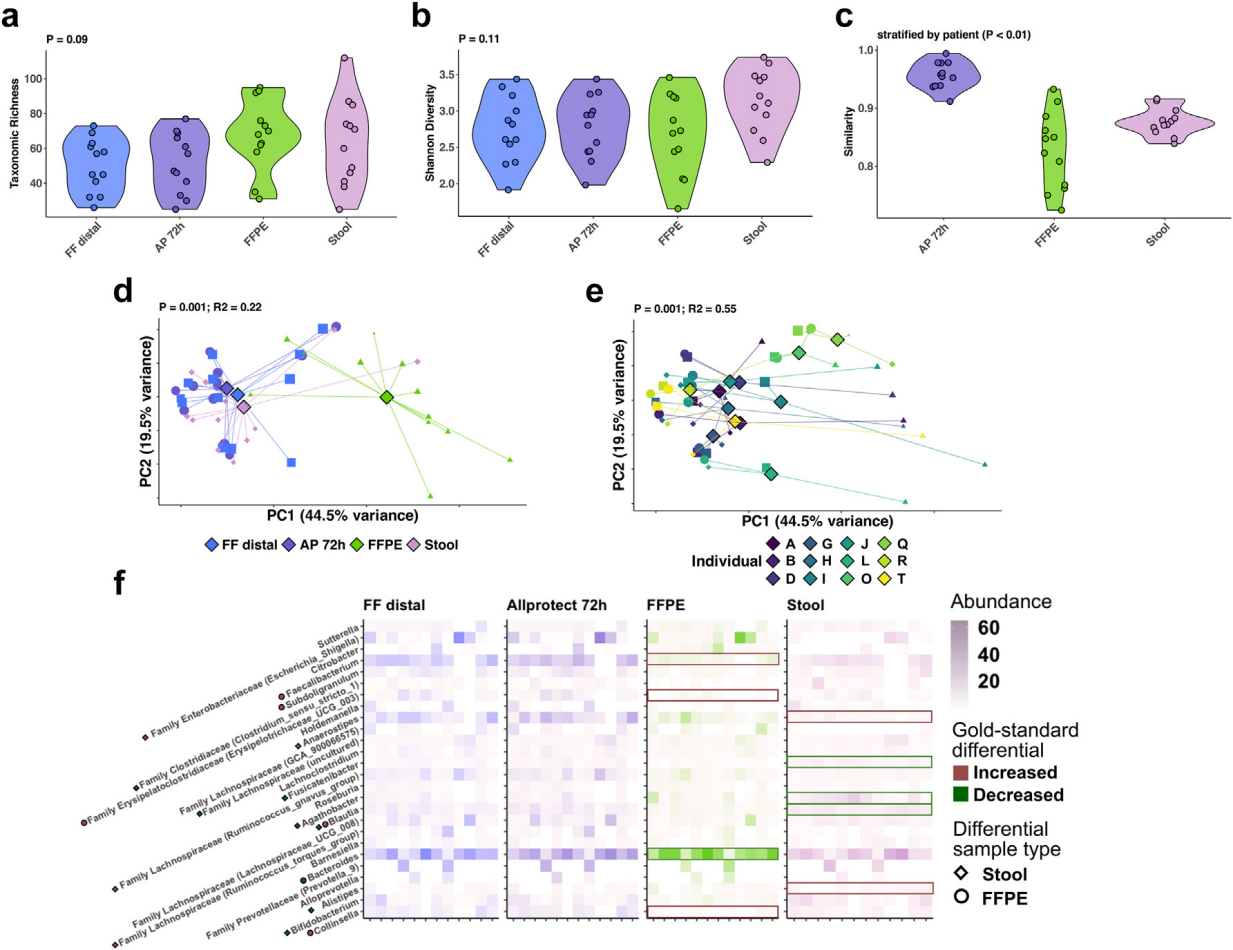
**Comparison of flash frozen (FF), AllProtect (AP), and formalinfixation with paraffin embedding (FFPE) tissue, with stool preserved using the OMNIgene®·GUT kit**. Violin plots (where each point represents an individual sample) showing comparison of: (a) taxonomic richness (Kruskal–Wallis P = 0.09); (b) Shannon diversity (Kruskal–Wallis P = 0.11); and (c) community similarity to flash frozen (distal) tissue (Kruskal–Wallis P < 0.01). Beta-diversity measures of bacterial community composition are shown by: (d) preservation method (PERMANOVA P = 0.001, R^2^ = 0.22), and (e) participant (PERMANOVA P = 0.001, R^2^ = 0.55). Differential taxa between FF, AP, and FFPE tissue, as well as stool are illustrated via a heatmap (f) where boxes highlight taxa identified as significantly differential between each sample type and flash frozen tissue (MaAsLin2 q < 0.05). Borderline differential taxa (q < 0.25) are annotated along the y axis by points according to sample type.

Next, we assessed how microbiota data acquisition compared between broad categories of sample types. We compared community composition of stool (as the archetype gut microbiota proxy, OMNIgene®•GUT sample) with tissue collected using gold standard (flash frozen), pragmatic (AllProtect® buffered), and standard healthcare processing (FFPE) methods. For this analysis all samples were rarefied at 1k reads. We included only those participants whose FFPE samples yielded >1k reads, facilitating microbiota comparison across the four experimental conditions of interest (n = 12/20). There was no significant difference in either taxonomic richness (Kruskal–Wallis P = 0.09) or Shannon diversity (Kruskal–Wallis P = 0.11) between preservation method or sample type (Fig. 3a–b). Beta-diversity analysis showed AllProtect®-preserved tissue more closely resembled flash frozen tissue than either FFPE (pairwise-Wilcoxon P = 4.4 × 10^−6^) or stool (pairwise-Wilcoxon P = 5.9 × 10^−6^). FFPE tissue was marginally more dissimilar to flash frozen tissue than stool (pairwise-Wilcoxon P = 0.045, Fig. 3c). Whilst using stool or FFPE instead of gold standard tissue processing was associated with changes to imputed microbiota signatures (PERMANOVA P = 0.001, R^2^ = 0.22; Fig. 3d), inter-individual differences were preserved, with participant still describing the majority of dissimilarity between microbiota compositions (PERMANOVA P = 0.001, R^2^ = 0.55; Fig. 3e). Differences between the microbial community of FFPE and stool, versus non-FFPE tissue were driven by differential abundance of obligate anaerobes; *Faecalibacterium* (MaAsLin2 q = 0.0001) and *Collinsella* (MaAsLin2 q = 0.006) were significantly less abundant in FFPE samples than flash frozen tissue, whilst *Anaerostipes* (MaAsLin2 q = 0.002) were significantly less abundant in stool. Several other anaerobic members of the Lachnospiraceae family were also differentially abundant between flash frozen tissue, and both stool and FFPE samples (Fig. 3f).

## Discussion

In this research, we demonstrate that nucleic acid preservative reagents can be used to store tissue suitable for mucosal microbiota analysis without a requirement for dry ice or liquid nitrogen. Comparable microbiota data was demonstrated even with variation in cold chain storage conditions, providing a pragmatic method for mucosal microbiota sampling at scale. We also demonstrate that, with limitations, FFPE tissue has utility for microbiota analysis. However, wax controls are important to allow for downstream correction of contamination from paraffin wax.

Large-scale, adequately-powered, multicentre studies with standardised sample collection are necessary to understand complex relationships between the gut mucosal microbiota, health and disease, with results that are generalisable to diverse patient populations.^17^^,^^35^^,^^36^ Stool samples offer a non-invasive means of interrogating microbial communities but are not necessarily reflective of the mucosal microbiota, that is the site of host–microbe interaction.^37^^,^^38^ Lacking spatial resolution, stool does not reflect longitudinal differences in microbial communities across the gastrointestinal tract. This lack of resolution is an important consideration in understanding disease biology where intra- and inter-individual variation in inflammatory activity across different segments of bowel is seen, such as in IBD. The gold standard of flash freezing tissue requires sample collection using either dry ice or liquid nitrogen and −80 °C freezer storage, with cold chain shipment where necessary. This necessitates sufficient cold chain infrastructure. Together with additional practical, safety, and cost implications, this gold standard method of sample collection is a major barrier to large, diverse and inclusive studies.

Whilst tissue is inherently reliant upon invasive sampling methods, the majority of IBD patients will have an endoscopic assessment of disease activity performed at the time of diagnosis.^34^^,^^39^, ^40^, ^41^ Procedures are also performed to assess disease activity during symptomatic flares and for colorectal cancer surveillance throughout the disease course.^34^^,^^39^, ^40^, ^41^ Multiple biopsy samples are routinely collected during these procedures to enable histopathological assessment of disease activity.^42^^,^^43^ This provides an opportunity to collect specimens suitable for microbiota research with limited additional burden to patients. Studying the mucosal microbiota with intestinal biopsy samples may identify unique signals that are more biologically relevant than those identified using stool that is reflective of the distal, luminal gut microbiota.

Compared to stool, biopsy specimens have a much lower microbial biomass. Despite this, we observed sufficient mapped bacterial sequencing reads from biopsy specimens to facilitate community analysis, suggesting endoscopic biopsies are a viable biospecimen for use in microbiota analyses. We found that stool and gold standard flash frozen biopsy tissue from the distal colon/rectum exhibited significant community dissimilarity, driven by differential abundance of obligate anaerobes. Importantly, stool is thought to reflect the distal gut microbiota.^44^ These differences highlight the limitation of using stool as a proxy for the mucosal microbiota, even when considering the distal colon as we did in this study.

We further tested differences between the various tissue collection methods. Previous studies have shown niche specification of the human gut microbiota across its ‘biogeographical axis’.^9^^,^^45^ We observed no difference between the most proximal and most distal sampling sites interrogated in this study, allowing us to systematically evaluate the effect of seven tissue preservation conditions. We saw no difference in microbiota signatures for biopsy samples collected and stored in three tissue preservative reagents (Allprotect®, DNA/RNA Shield™, RNAlater®), compared to gold standard flash frozen samples. Thus, each of these preservative solutions provides a feasible alternative to flash freezing samples. Importantly, neither duration of short-term storage at 4 °C, or longer-term storage at −20 °C (instead of −80 °C) impacted community structure. Our findings will support pragmatic future study design, enabling collection of samples in hospitals with differing resource for tissue handling and cold chain storage, including e.g. 4 °C shipping to central laboratories, out of hours collection, next day or post weekend processing.

Participants undergoing colonoscopy as part of routine healthcare assessments will typically have multiple biopsies taken during procedures.^42^^,^^43^ These biopsy samples are processed to generate FFPE tissue blocks for histopathological examination. The Royal College of Pathologists (United Kingdom) recommend that primary FFPE specimens are retained for a minimum of 30 years.^16^ Thus, there is an extensive repository of tissue from IBD patients within the NHS that represents a largely untapped resource for microbiota research.

In this study, library sizes of FFPE tissue samples were significantly lower than non-FFPE tissue. From raw data, we excluded samples with less than 1000 mapped bacterial reads. Applying this exclusion criterion led to loss of seven FFPE samples from our analysis. One FFPE sample failed to amplify during sequencing. Due to differences in library size between samples, analyses were performed using rarefied data. Although the removal of large quantities of reads from non-FFPE tissue may affect measurable richness and diversity of samples,^46^ even at this comparably low read depth, rarefaction curves were approaching an asymptote, suggestive of sufficient sampling depth. Furthermore, rarefying remains a widely used and accepted approach to normalising data within microbial ecology.^47^ In the remaining samples, we were able to demonstrate comparable alpha-diversity to other tissue samples. Although community composition of FFPE differed from other tissue samples, this divergence was also seen with stool. Despite this compositional difference, it remained possible to impute a distinct individual microbiota signature using FFPE.

The use of FFPE does have limitations. Due to paraffin embedding of samples, nucleic acids were extracted using a dedicated FFPE sample extraction kit incorporating additional deparaffinisation steps. The use of a different DNA extraction kit may have introduced systemic bias, impacting conclusions drawn about (dis)similarity of FFPE to other tissue samples.^48^

Formalin fixation may lead to cross-linking of nucleic acids and proteins that blocks DNA polymerases during library amplification.^49^ The FFPE DNA extraction kit used in this study incorporates an additional heat incubation step, intended to thermally disrupt these cross-links. DNA cross-linking may still have contributed to the observed differences in library size between FFPE and non-FFPE samples in this study. Previous studies indicate that longer formalin exposure time is associated with greater DNA degradation and differential efficiency of sequencing.^50^ The length of time FFPE samples remained in formalin varied in this study (Supplementary Table S4). Although we observed no correlation between formalin time and library size (P = 0.47; R_2_ = 0.03, Supplementary Figure S11), our data are based on a small sample size. Formalin may also cause hydrolytic deamination of cytosine to uracil leading to C:G > T:A base substitutions during sequencing.^51^^,^^52^ This may have impacted mapping of reads from FFPE samples. Further optimisation of FFPE DNA extraction protocols, such as routine incorporation of uracil-DNA glycosylase repair steps and computational removal of other deamination artefacts is needed to maximise the utility of FFPE.^51^^,^^52^

We processed tissue samples into FFPE blocks within three days and performed subsequent nucleic acid extraction within one month of sample collection. It is not possible to draw conclusions about the utility of long-term archived FFPE samples relative to recently created FFPE samples as a tool to study the mucosal microbiota from this data. Nevertheless, the repository of FFPE samples that exists within healthcare settings, including samples collected at clinically significant timepoints such as time of first diagnosis (prior to medical treatment), during periods of disease flare and remission is unmatched by any prospective study. Whilst we demonstrate limitations in the use of FFPE, interrogating the gut mucosal microbiota of these historical samples in large numbers may unlock potentially significant biological insights.

### Conclusions

Tissue preservative solutions give comparable results to flash frozen tissue, including comparable library size, richness and community composition. Our data indicate that more pragmatic approaches to tissue collection and storage are a feasible alternative to flash freezing that will facilitate large-scale research evaluating the mucosa-associated microbiota and help ensure appropriate statistical power and generalisability of future research.

## Contributors

N.J.W., C.J.S. and C.A.L. conceptualised and designed the study. M.D., R.L., N.T., and N.J.W. recruited participants and collected clinical metadata. A.K., R.A.S. and C.A.L. performed colonoscopy procedures to obtain research biopsy specimens. M.D., H.W., N.T., J.A.D. and N.J.W. received and processed participant samples. G.R.Y. led the statistical analysis with input from A.C.M., N.T., H.W. and N.J.W. Data were accessed and verified by G.R.Y., N.J.W., H.W., and C.J.S. N.J.W. and H.W. wrote the initial draft of the manuscript with input from G.R.Y., C.J.S. and C.A.L. All other authors (M.D., N.T., D.A., A.C.M., T.A., J.A.D., K.F., A.H., V.H., P.M.I., C.J., N.A.K., S.L., C.W.L., R.L., T.L., J.O.L., J.R.M., M.P., N.P., N.J.P., T.R., J.S., K.W., R.W., A.K., L.J.D., R.A.S., and N.M.) provided critical review and comment on the study design and approved the final draft of the manuscript.

## Data sharing statement

De-identified data will be made available to others for meta-analysis upon request and review through IBD-RESPONSE Study Management Group discussion and signing of a data access agreement. Requests for access to data should be made to the senior and first authors via the corresponding email given. Raw sequencing data is available at the European Nucleotide archive under project accession PRJEB74359.

## Declaration of interests

None to declare.
