# Supplementary material for: Evaluation of intestinal biopsy tissue preservation methods to facilitate large-scale mucosal microbiota research
Source: eBioMedicine. 2024 Dec 31;112:105550. doi: 10.1016/j.ebiom.2024.105550 (PMC11751561; doi:10.1016/j.ebiom.2024.105550)

SUPPLEMENTARY TABLES AND FIGURES

Evaluation of intestinal biopsy tissue preservation methods to facilitate large-scale mucosal microbiota research

Table of contents

Supplementary table 1	…………	Page 3
Supplementary table 2	…………	Page 4
Supplementary table 3	…………	Page 5
Supplementary table 4	…………	Page 5
Supplementary table 5	…………	Page 5
Supplementary figure 1	…………	Page 6
Supplementary figure 2	…………	Page 7
Supplementary figure 3	…………	Page 8
Supplementary figure 4	…………	Page 8
Supplementary figure 5	…………	Page 9
Supplementary figure 6	…………	Page 10
Supplementary figure 7	…………	Page 11
Supplementary figure 8	…………	Page 12
Supplementary figure 9	…………	Page 13
Supplementary figure 10	…………	Page 14
Supplementary figure 11	…………	Page 15

Supplementary table 1: Extracted DNA concentrations of participant samples.

	DNA concentration (ng/µL)									
	Stool		Non-FFPE tissue							FFPE tissue
Participant	Omnigene	DNA/RNA Shield	Flash frozen proximal	Allprotect 4ºC 24h	Allprotect 4ºC 72h	Allprotect 4ºC 1 month	RNAlater	DNA/RNA Shield	Flash frozen distal	
A	85·7	94·1	70	237	128	143	86·1	16·8	215	8·8
B	84·6	63·9	370	184	185	138	224	27.2	322	29.8
C	72·2	44·3	108	151	205	188	250	23·2	209	53·4
D	37·9	74·9	194	192	124	131	206	20·4	147	24·5
E	64	46·8	224	151	121	175	193	20·7	220	54·1
F	21·3	51·5	54·5	43·9	26·7	15·9	51·4	9·9	18.2	68.9
G	95·5	37·7	106	88·3	122	34·8	86·7	19·3	50·8	58·4
H	258	94·6	133	131	103	108	72·1	21·5	79·4	24·1
I	31·4	35·4	85·1	83·7	132	75.2	101	11·7	98·9	5·8
J	27	286	434	342	422	296	279	42	280	74·8
K	14	186	311	210	146	322	380	46·9	201	131
L	264	64·8	299	253	203	208	220	20	340	117
M	892	26·4	159	93·2	17·1	189	252	6·3	61·2	92·4
N	15·7	62	403	339	316	357	333	56·3	410	98·2
O	18·5	309	137	251	220	149	71	26·5	115	302
P	12·4	139	184	208	165	211	210	30·2	310	94·8
Q	10·2	38·1	195	221	258	223	92·1	33·7	187	131
R	540	201	206	160	139	165	206	15·3	204	154
S	8·9	180	327	186	274	265	277	31·9	299	127
T	190	40·7	161	252	127	152	147	42	237	58·2
**Mean**	137·2	103·8	208	188·9	171·7	177·3	186·9	26·1	200·2	85·4

Supplementary table 2: Contaminant OTUs identified and removed from the dataset using decontam package in R.

Genus	Total reads	Total Prevalence (Control)	Prevalence % (Control)	Total Prevalence (Sample)	Prevalence % (Sample)
Pseudonocardia	16	3	7·5	1	0·5
Pseudomonas	108	5	12·5	16	8
Pseudomonas	811	6	15	29	14·5
Enhydrobacter	2219	9	22·5	33	16·5
Acinetobacter	1500	8	20	34	17
Pseudomonas	36	1	2·5	7	3·5
Microvirga	11	0	0	3	1·5
Flavobacterium	50	3	7·5	3	1·5
Sphingobacterium	28	1	2·5	2	1
Sphingobacterium	22	1	2·5	3	1·5
Thiothrix	25	1	2·5	3	1·5
Chryseobacterium	118	4	10	11	5·5
Paracoccus	788	5	12·5	19	9·5
Tabrizicola	60	4	10	13	6·5
Sphingomonas	240	6	15	20	10
Family Microbacteriaceae	208	4	10	12	6
Kocuria	1175	8	20	35	17·5
Georgenia	66	3	7·5	5	2·5
Neisseria	448	7	17·5	22	11
Tepidimonas	117	6	15	16	8
Cupriavidus	40	2	5	9	4·5
Family Neisseriaceae (uncultured)	1251	8	20	31	15·5
Micrococcus	216	7	17·5	27	13·5
Williamsia	258	7	17·5	29	14·5
Luteipulveratus	2324	9	22·5	35	17·5
Streptococcus	1868	8	20	29	14·5
Staphylococcus	3034	8	20	35	17·5
Bacillus	67	5	12·5	5	2·5

Supplementary table 3: Median (IQR) Good's coverage by sample type. P values calculated by comparison of mean paired sample GC score at each rarefaction depth to unrarefied, raw reads.

	Raw reads	Rarefied @ 4.6k reads	P value	Rarefied @ 1k reads	P value
**Negative**	0·852 (0·828 - 0·87)	0·852 (0·828 - 0·87)	1	-	-
**Wax**	0·919 (0·904 - 0·931)	0·919 (0·904 - 0·931)	1	-	-
**FFPE**	0·978 (0·892 - 0·986)	0·974 (0·892 - 0·979)	0·014	0·958 (0·948 - 0·964)	<0·0001
**Tissue**	0·998 (0·997 - 0·999)	0·98 (0·973 - 0·985)	<0·0001	0·959 (0·953 – 0·970)	<0·0001
**Stool**	0·994 (0·993 - 0·997)	0·982 (0·975 - 0·987)	<0·0001	0·949 (0·943 - 0·965)	<0·0001

Supplementary table 4: Key dates for stool sample collection and receipt, and tissue collection (date formalin fixation started) and subsequent tissue removal from formalin.

Sample ID	Stool date collected	Stool date received (procedure date)	Date formalin fixation started	Date removed from formalin
A	10.04.22	12.04.22	12.04.22	13.04.22
B	11.04.22	12.04.22	12.04.22	13.04.22
C	10.05.22	10.05.22	10.05.22	10.05.22
D	16.05.22	17.05.22	17.05.22	19.05.22
E	26.06.22	28.06.22	28.06.22	29.06.22
F	10.06.22	10.06.22	10.06.22	11.06.22
G	22.06.22	24.06.22	24.06.22	27.06.22
H	03.07.22	05.07.22	05.07.22	05.07.22
I	01.07.22	05.07.22	05.07.22	05.07.22
J	09.07.22	12.07.22	12.07.22	13.07.22
K	11.07.22	12.07.22	12.07.22	13.07.22
L	14.07.22	15.07.22	15.07.22	16.07.22
M	22.07.22	26.07.22	26.07.22	27.07.22
N	10.09.22	13.09.22	13.09.22	14.09.22
O	18.08.22	23.08.22	23.08.22	24.08.22
P	22.08.22	23.08.22	23.08.22	24.08.22
Q	22.08.22	23.08.22	23.08.22	24.08.22
R	07.09.22	09.09.22	09.09.22	10.09.22
S	19.09.22	20.09.22	20.09.22	21.09.22
T	18.09.22	20.09.22	20.09.22	21.09.22

Supplementary table 5: Compositional dissimilarity matrix (p values) (ANOSIM).

	Stool	Tissue	FFPE	Wax	Negative
**Stool**		0·351	0·001	0·001	0·003
**Tissue**	0·351		0·002	0·001	0·001
**FFPE**	0·001	0·002		0·001	0·046
**Wax**	0·001	0·001	0·001		0·001
**Negative**	0·003	0·001	0·046	0·001	

Supplementary figure 1: Sequencing stats of key sample types following removal of potential contaminant taxa. (a) Density plots extend to the full range of library sizes with each point representing an individual sample (FFPE tissue samples denoted 'FFPE' with non-FFPE tissue samples denoted 'tissue'). (b) Rarefaction curve shows sufficiency of sampling depth with each line representing an individual sample. 4.5k rarefaction threshold indicated with dashed grey line on each panel.

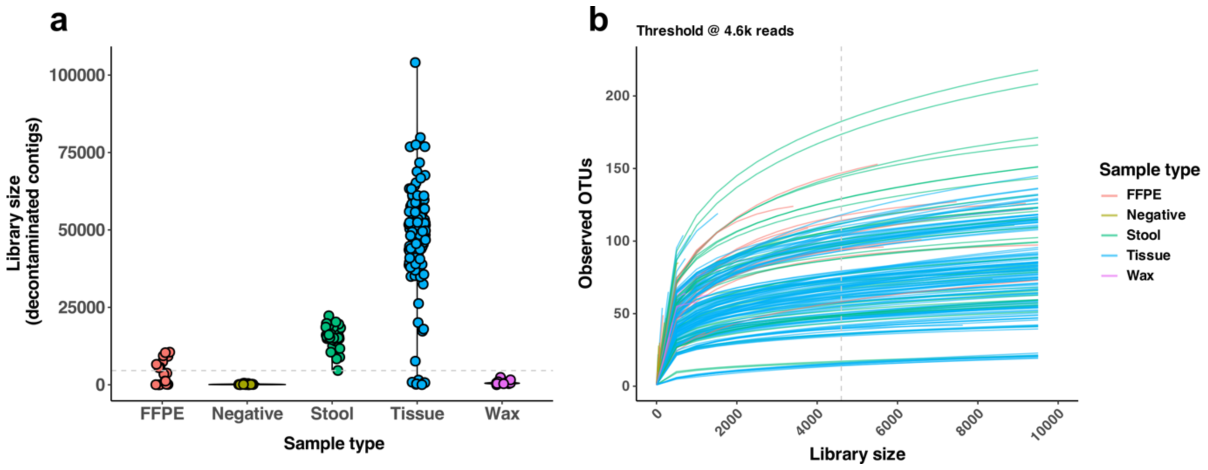

Supplementary figure 2: Good's coverage for raw (unrarefied) and rarefied (at 4.6k reads) samples by type (a), as well as raw (unrarefied) and rarefied (at 4.6 and 1k reads) for all samples involved in FFPE comparison analysis (b).

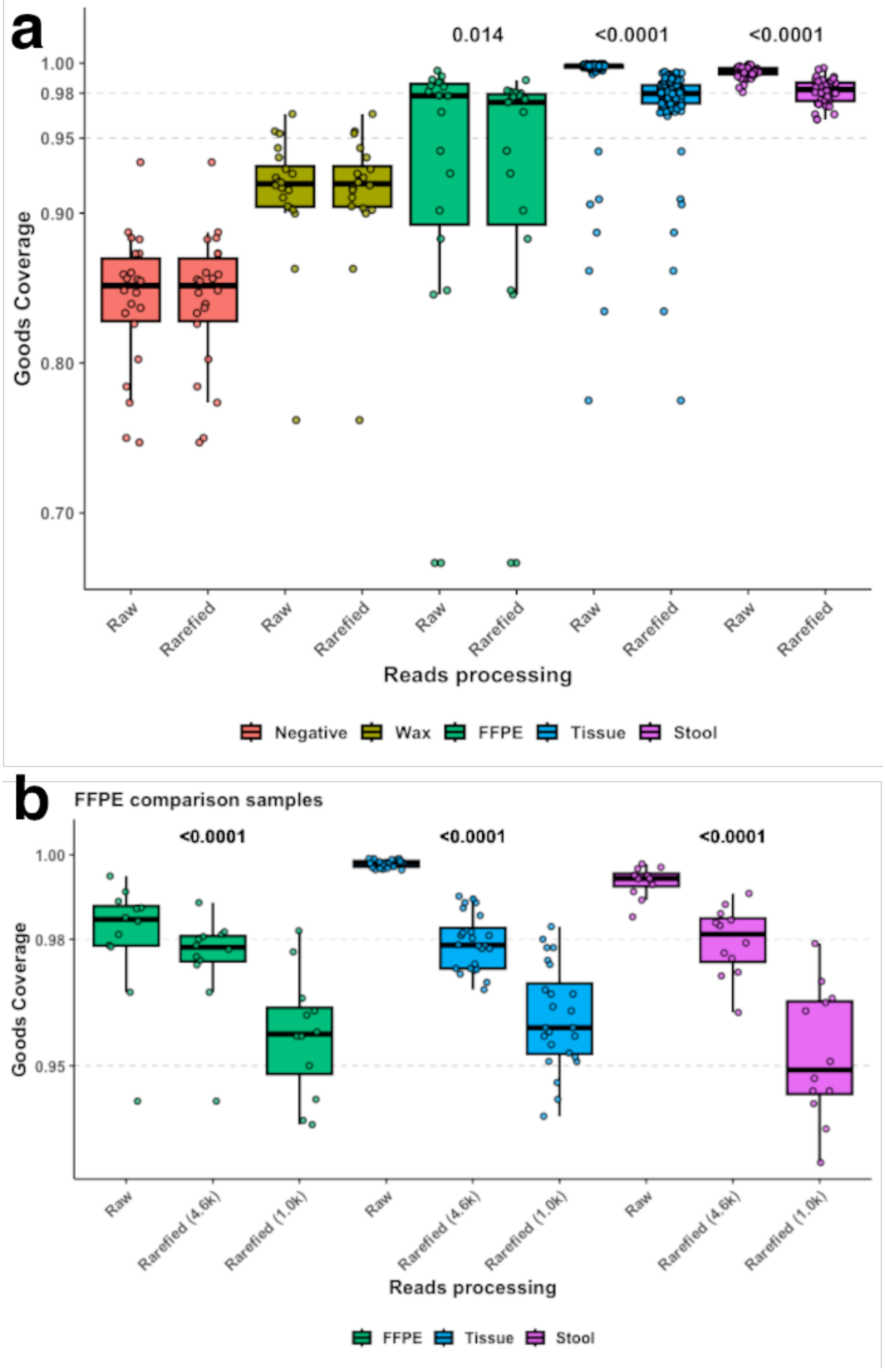

Supplementary figure 3: Paired distances from each anal verge of most proximal and distal intestinal biopsy samples collected from each participant.

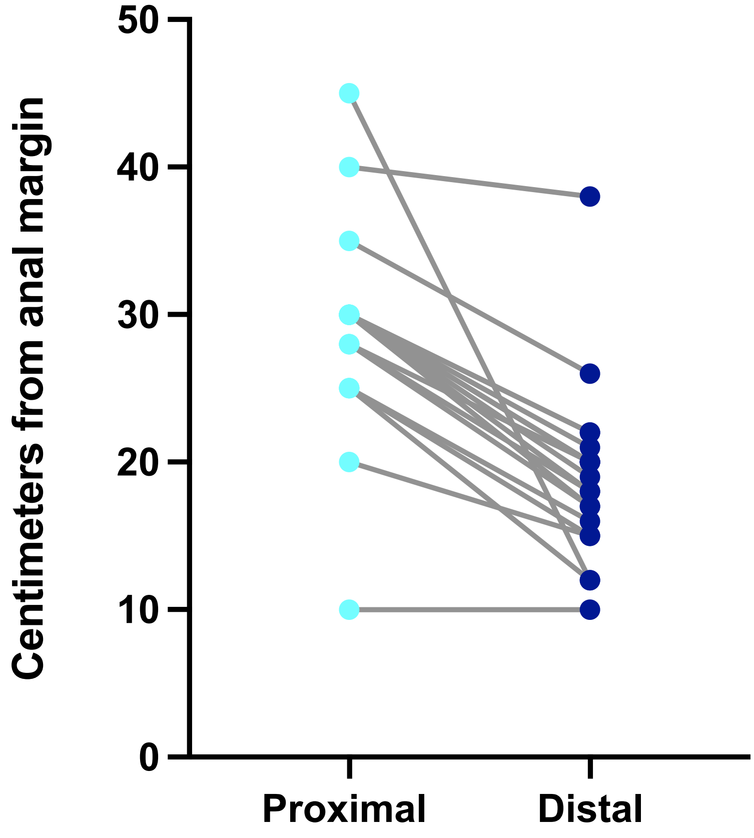

Supplementary figure 4: Histogram showing the number of days that tissue specimens remained in formalin prior to paraffin embedding.

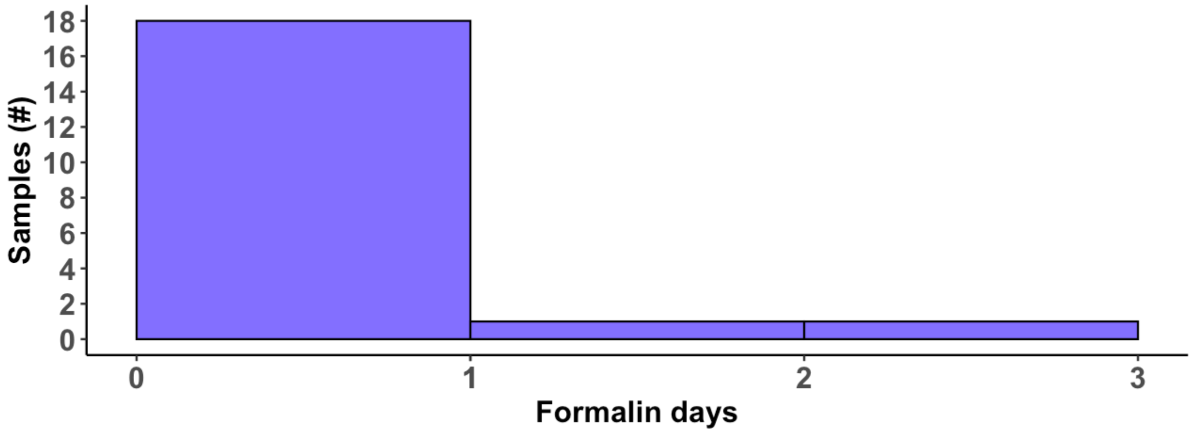

Supplementary figure 5: Density plots showing library sizes for participant (all tissue and stool conditions) versus control samples after performing the decontam procedure to eliminate contaminant taxa (n=28). Each point represents an individual sample.

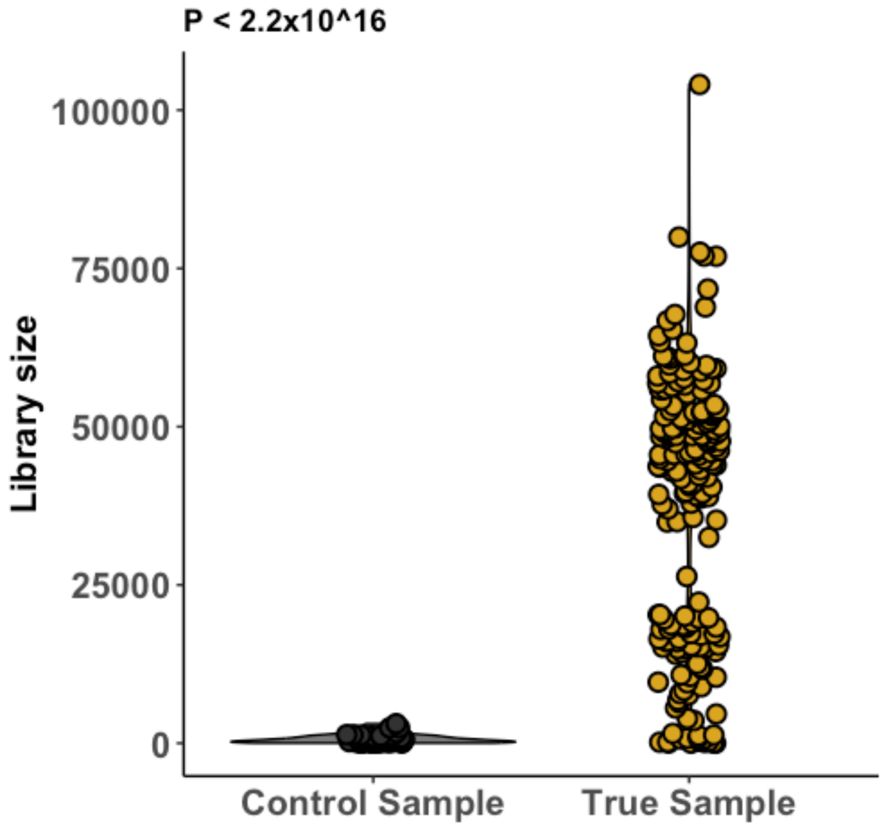

Supplementary figure 6: Principal components analysis (PCoA) plot of community composition for participant samples (FFPE, tissue, stool) and control samples (kit negative and wax). Each point represents an individual sample, coloured by sample type and sized by library size. All samples are connected to a centroid (bold outline) representing the average Euclidean position of that sample type.
NB. 'Tissue' denotes all non-FFPE tissue samples from participants.

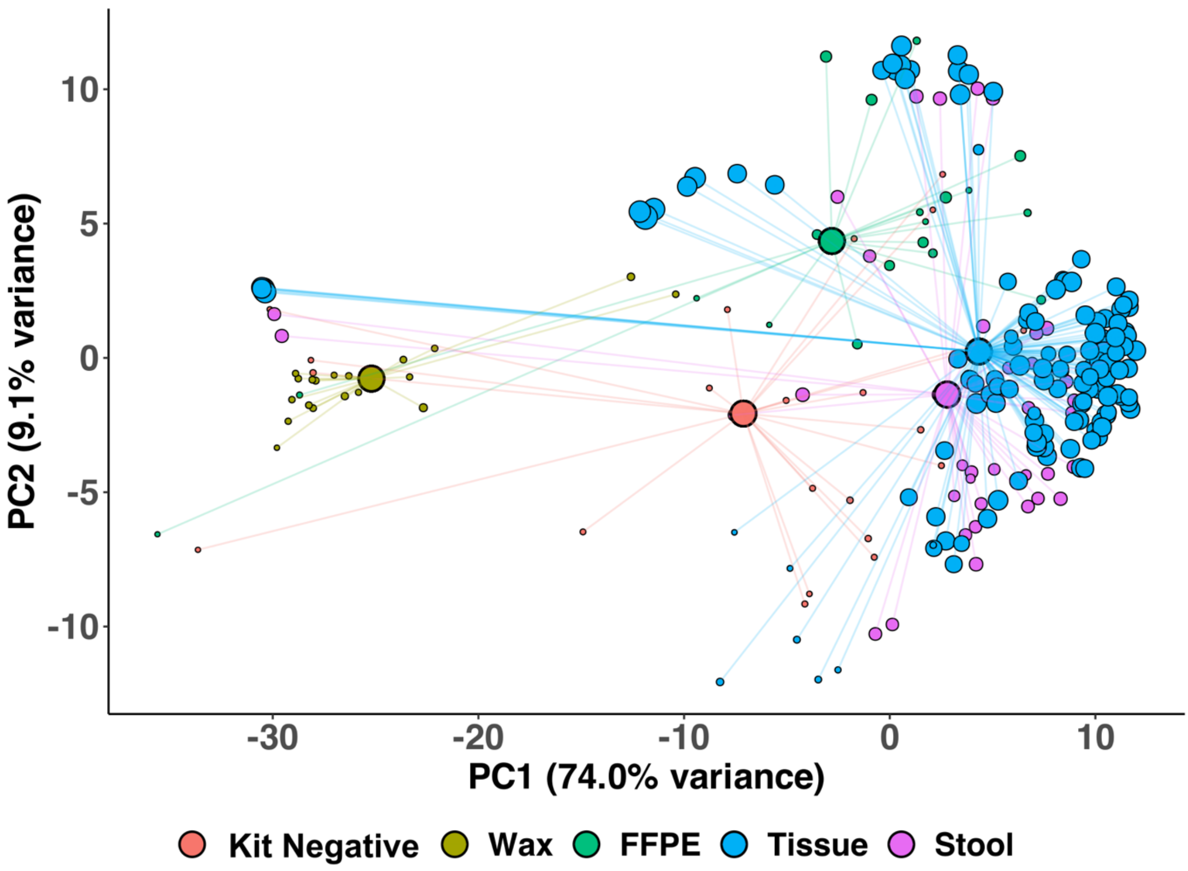

Supplementary figure 7: Heatmap illustrating taxonomic composition (minimum mean sample abundance 50%) of flash frozen and reagent-preserved tissue samples, stratified by preservation method. (Abbreviations: FF, flash frozen)

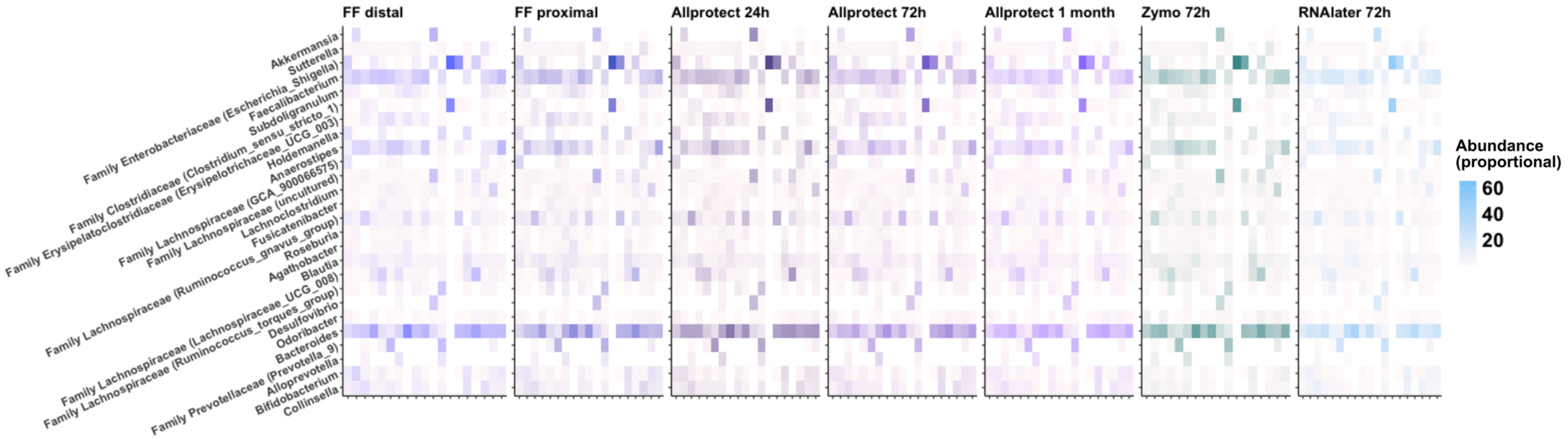

Supplementary figure 8: Library size comparison for participant (FFPE) vs control (wax) samples after performing the Decontam procedure to eliminate contaminant taxa (n=28).

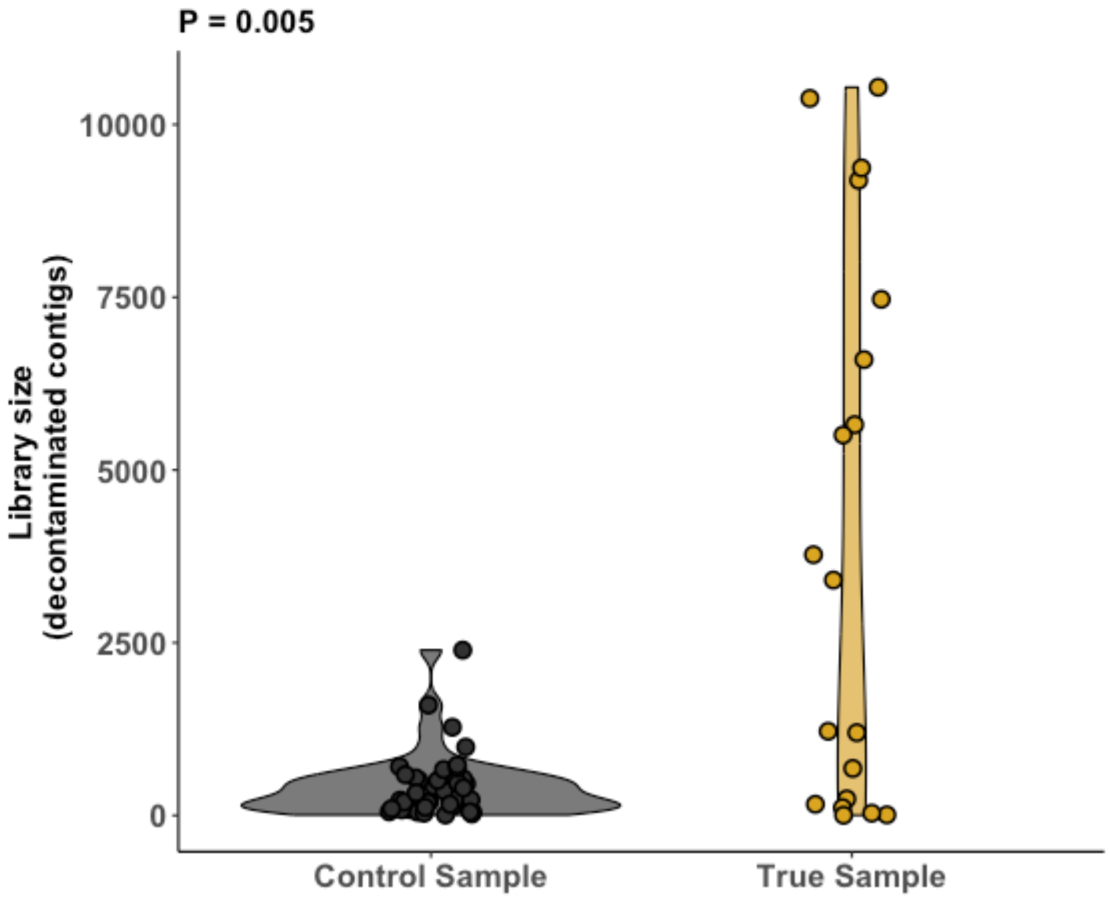

Supplementary figure 9: Comparison of wax negative control ('wax'), FFPE tissue ('FFPE'), and flash frozen (FF) tissue (distal site, tissue condition 8 as the comparator) unrarefied library sizes.

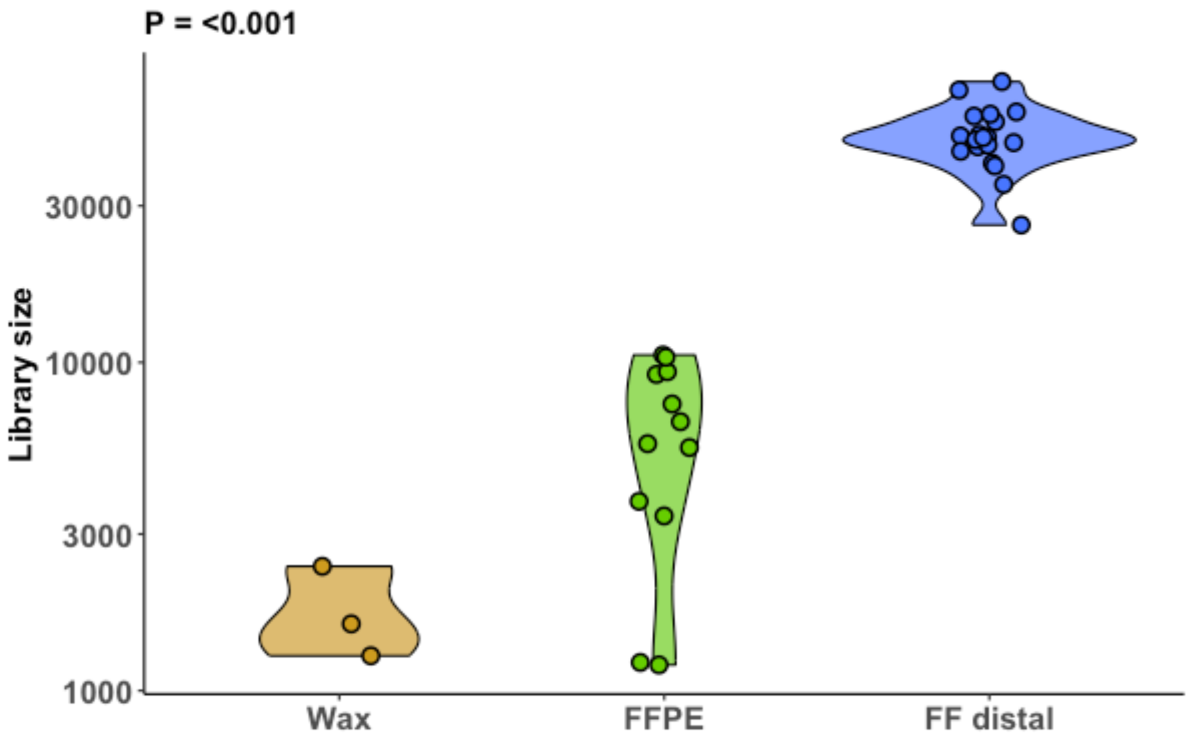

Supplementary figure 10: Comparison of flash frozen tissue (distal sample site) and stool samples collected using two commercially-available sample collection kits containing nucleic acid preservative buffer, showing library sizes (a), alpha diversity as taxonomic richness (b), alpha diversity as Shannon index (c), and a principal components analysis plot of community composition (beta-diversity) for the two stool conditions.

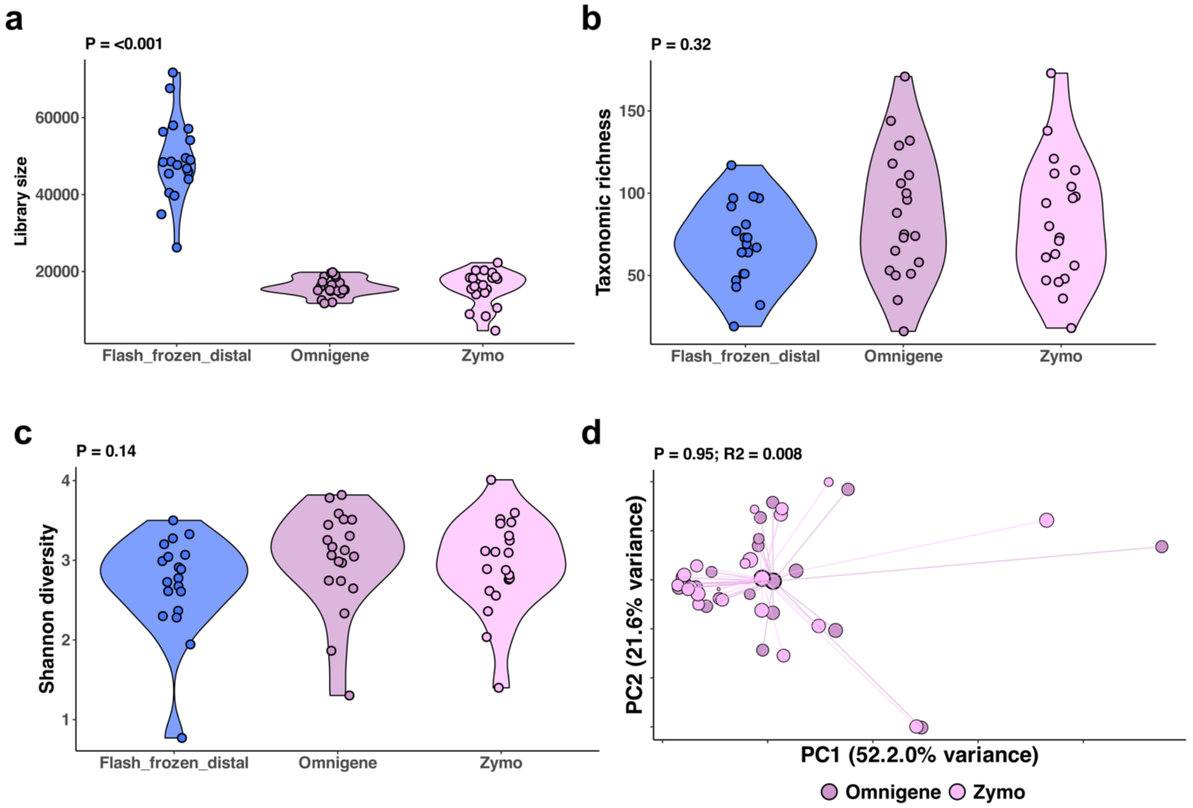

Supplementary figure 11: Library sizes of FFPE tissue samples by time spent in formalin (days).

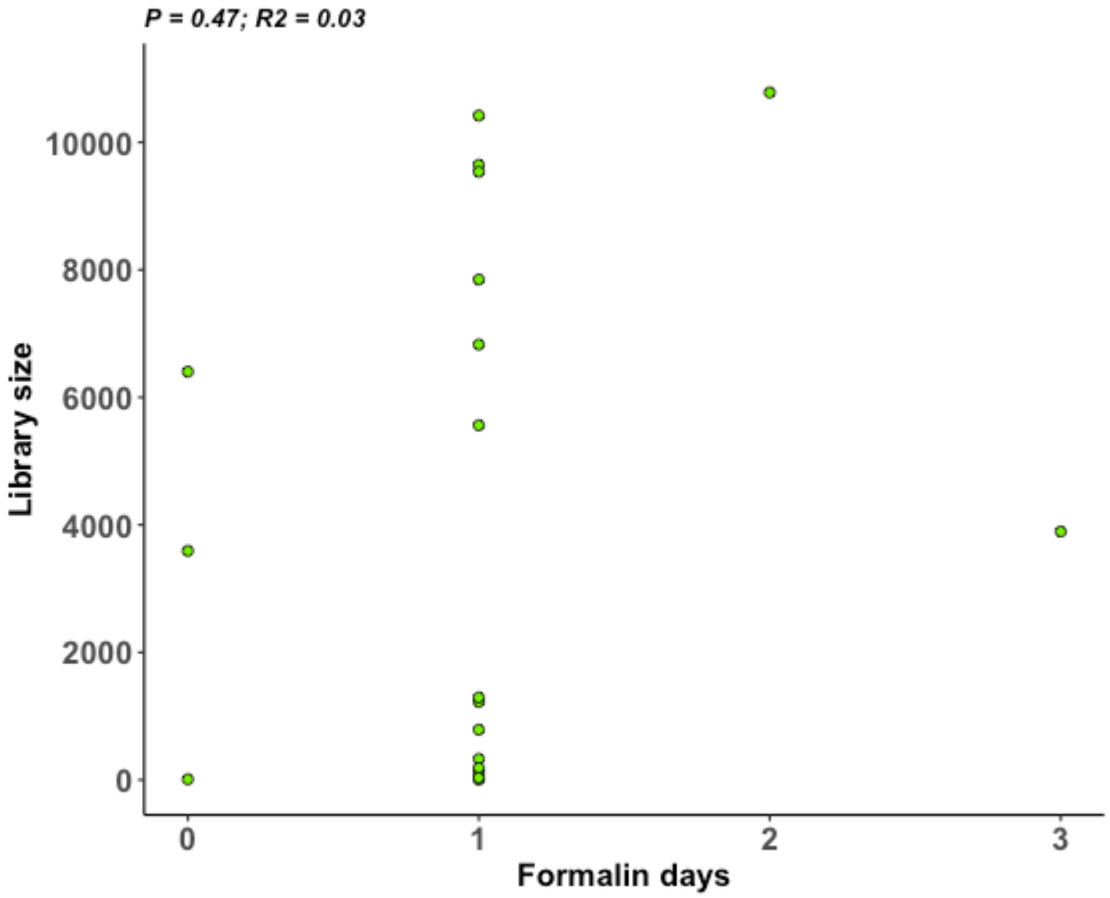

Supplement: Supplementary Figures and Tables [file mmc1.pdf]
